# Comparison of clear and narrow outcomes on testosterone levels in social competition^☆^

**DOI:** 10.1016/j.yhbeh.2016.05.016

**Published:** 2017-06

**Authors:** Yin Wu, Christoph Eisenegger, Samuele Zilioli, Neil V. Watson, Luke Clark

**Affiliations:** a Behavioural and Clinical Neuroscience Institute, Department of Psychology, University of Cambridge, Cambridge, UK; b Research Center for Brain Function and Psychological Science, Shenzhen University, Shenzhen, China; c Faculty of Psychology, University of Vienna, Vienna, Austria; d Department of Psychology, Wayne State University, Detroit, USA; e Department of Psychology, Simon Fraser University, Burnaby, Canada; f Centre for Gambling Research at UBC, Department of Psychology, University of British Columbia, Vancouver, British Columbia, Canada

**Keywords:** Near-miss, Decision-making, Dominance, Hormone, Cortisol, Dual-hormone hypothesis

## Abstract

A contribution to a special issue on Hormones and Human Competition.

Social competition is associated with marked emotional, behavioral and hormonal responses, including changes in testosterone levels. The strength and direction of these responses is often modulated by levels of other hormones (e.g. cortisol) and depends on psychological factors – classically, the objective outcome of a competition (win vs. loss) but also, hypothetically, the closeness of that outcome (e.g. decisive victory vs. close victory). We manipulated these two aspects of a social contest among male participants (N = 166), to investigate how testosterone and affect fluctuated as a function of clear vs. narrow wins and clear vs. narrow losses. We found that losing a competition by a small margin (a narrow loss) was experienced as more pleasant than a clear loss. Among individuals with higher levels of basal cortisol, winning the competition by a narrow margin was associated with a *decrease* in testosterone levels. These findings are discussed within the framework of the status instability hypothesis and the growing literature on how situational and physiological factors modulate testosterone reactivity to social contests.

## Highlights

• A competition task was developed to compare clear/narrow victories and defeats.
• Narrow victories reduced testosterone among individuals with higher basal cortisol.
• Narrow defeats were experienced as more pleasant and increased motivation to play.

## 1 Introduction

Competition is the prevailing mean for determining status within both human and non-human social hierarchies (Magee and Galinsky, 2008, Sapolsky, 2004). Acquisition of status can lead to remarkable emotional responses to competitive outcomes, such as joy after a victory (a gain of status) and frustration after a defeat (and loss of status). Social contests are also associated with hormonal fluctuations, primarily with regard to testosterone levels. According to the Challenge Hypothesis (Archer, 2006), testosterone levels rise during periods when competitive and aggressive behaviors are common, and drop during periods of social stability. These fluctuations depend further on the outcome of social contests, such that winners tend to experience an increase in testosterone compared to losers. This observation has been labelled the “winner–loser effect” and is central to the Biosocial Model of Status (BMS) (Mazur, 1985, Mazur and Booth, 1998), which highlights the adaptive consequences of outcome-related testosterone change. According to the BMS, winning a competition is associated with a rise in social status, and testosterone increases may serve to promote competitive and aggressive behavior aimed at defending and maintaining one's new position. On the other hand, losing a contest may lower social status, and testosterone decreases may promote submissive behaviors that serve to avoid further loss of status or physical harm.

While these basic tenets of the BMS have been replicated numerous times (for a review, see Carré and Olmstead, 2015, Hamilton et al., 2015), an increasing number of experiments indicate that a more nuanced account is required, to explain various situational and psychological variables that can give rise to not only null results but even a full inversion of the classic winner effect. During competitions characterized by close outcomes (e.g. barely winning or barely losing), the winner-loser effect has been seen to reverse, such that losers showed *increased* testosterone relative to winners (Zilioli et al., 2014). Zilioli and colleagues argue that testosterone increases after unstable losses and decreases after unstable wins, a phenomenon termed the *status instability hypothesis* and corroborated by other data (Oliveira et al., 2013, Oliveira et al., 2014). By this account, close or uncertain outcomes render the status hierarchy unstable and in such circumstances, status-seeking behaviors mediated by increased testosterone could enable lower status individuals to grasp an opportunity to enhance their status. Conversely, reduced testosterone after a close victory may promote the avoidance of further contests as a strategy to protect one's vulnerable high-status position from being lost in the unstable environment. While appealing, the earlier experiment by Zilioli et al. (2014) only compared close contests, and did not use a fully-factorial design comparing close wins and losses against decisive wins and losses. Zilioli et al. (2014) also tested female samples exclusively, and hence it is unclear if these findings generalize to the larger literature on male competition.

The aim of the present study was to investigate testosterone responses to winning and losing, where the closeness between winners and losers was manipulated. We predicted that the outcome of the competition (win vs. loss) would interact with the closeness of the outcome (narrow vs. clear) in determining the change in testosterone levels. Specifically, the status instability hypothesis predicts that testosterone levels would increase in clear winners and narrow losers, and decrease in clear losers and narrow winners (Zilioli et al., 2014). In light of recent studies showing how testosterone responses to competition outcomes can further depend on basal cortisol levels, we also obtained pre-competition salivary cortisol samples (Edwards and Casto, 2015, Zilioli and Watson, 2012). Pre-competition cortisol levels were found to be negatively associated with testosterone change in both winners and losers, following a laboratory competition procedure (Mehta and Josephs, 2006), and similar findings have been shown in field observations of athletic competitions (Edwards and Casto, 2015).

We also obtained subjective ratings of affect and motivation, to extend a further line of research showing that a narrow loss can elicit a stronger subjective motivation to play than categorical victories (Clark et al., 2009). For example, in professional basketball games, teams that were slightly behind at halftime were more likely to win the match than the teams that were slightly ahead (Berger and Pope, 2011). Similar effects are well established in gambling behavior, in which “near-miss” outcomes that just fall short of a significant payout are associated with increased motivation to play and more persistent gambling (Clark et al., 2009, Cote et al., 2003). These results indicate that emotional responses to social contests do not scale with outcome in a simple monotonic fashion (whereby losers would always feel more negative than winners). We predicted that narrow losses would increase subjective ratings of the desire to play the game again, a measure of motivation.

Our social competition task was a modified version of the 2-player Tetris competition developed by Zilioli and Watson, 2012, Zilioli and Watson, 2014 and Zilioli et al. (2014). Pairs of undergraduate male participants played against one another in a 15 minute contest, using two computer terminals in adjacent testing rooms. The competitor who scored higher (by completing the most lines) was designated the winner and received an additional prize. In reality, game outcomes were pre-determined such that participants were randomly assigned to the four conditions, to enable testosterone changes to be disambiguated from differences in effort or true performance. We modified the original procedure to experimentally manipulate the closeness of the victory or defeat, such that in some pairs, one player would experience a resounding victory by a large points distance – henceforth a *clear win*, contrasting with their opponent sustaining a *clear loss*. In other pairs, the scores were extremely close, representing a *narrow win* and *narrow loss*.^1^ This mimics many real-world competitions that involve a continuous dimension of “distance” between competitors. We reinforced our four outcome types by presenting verbal feedback to participants *during* the Tetris game, in the form of a series of on-screen SMS messages from the experimenter (e.g. for narrow losers “Keep going, you are slightly behind!”). Prior to the experiment reported here sampling testosterone levels, we piloted the modified Tetris game in 87 participants to confirm differential effects of outcome closeness on subjective ratings (i.e. affect following the outcome, and motivation to play, see Supplementary material). In the present study, we tested male participants exclusively, as previous research on the winner-loser effect has shown stronger effect sizes for testosterone change in males than females (Carré and Olmstead, 2015, Carré et al., 2013).

## 2 Methods

### 2.1 Participants

One hundred and sixty-six male volunteers (mean age = 23.2, SD = 3.27; age range = 19–33) were recruited using advertisements around the university. Seventy percent identified as White/Caucasian, 22% as Asian, 8% as ‘Other’. Volunteers attended a single testing session, where they completed the Tetris game (15 min), post-experiment questionnaires, and provided two saliva samples. Participants attended test sessions in pairs, after selecting a test slot via the laboratory website. Thus generally, participants did not know each other prior to arrival, as this was discouraged on the website. The opponents met each other upon arrival at the lab, to reinforce the competitive element. The study was conducted in accordance with the Declaration of Helsinki and was approved by University of Cambridge Human Biology Research Ethics Committee. Written informed consent was obtained from all participants. Participants were reimbursed £12 (~ US$18) for participation.

### 2.2 Two-player Tetris game

The competitive task was adapted from the Tetris game previously used by Zilioli and Watson, 2012, Zilioli and Watson, 2014. Tetris is a speeded puzzle game in which different two-dimensional shapes drop down the screen, and must be rotated and fitted together into rows. If a player “fills” an entire line with no spaces, that line disappears to create more space for the falling blocks. As the game unfolds, the speed at which the blocks drop increases, resulting in steadily increasing difficulty and cognitive effort by the player. Each participant was led to believe that he was competing against the other participant via two linked computers. Unbeknownst to the participants, the outcome of the task was manipulated, such that winning and losing conditions were pre-assigned rather than determined by performance. An important feature of this variant of Tetris was that if the screen filled with blocks, the game did not terminate (as in the classic game) but rather the screen would shift the blocks down, allowing all participants to continue for the required 15 minute period, regardless of their prior experience level or ability. After 15 min of play, the message “you win!” on a colorful background was displayed on the winner's screen, while the loser's screen displayed “you lose!” on a drab background.

We manipulated the closeness of scores between winners and losers by imposing two features. First, immediately following the outcome display (i.e. the “you win!”/“you lose!” message), both the participant's and opponent's scores were presented. The participant's score was necessarily veridical, but the opponent's score was manipulated in order to pre-configure the four outcome types. In the clear win condition, the opponent scored 30% of the participant's score (e.g. participant vs. opponent: 1436 vs. 431). In the clear loss condition, the opponent scored 1.7 times of the participant's point (e.g. 1436 vs. 2441). In the narrow win condition, the opponent scored 11 points less than the participant (e.g. 1436 vs. 1425). In the narrow loss condition, the opponent scored 11 points more than the participant (e.g. 1436 vs. 1447). Second, throughout the competition, participants were presented with scripted messages in the upper right corner of the Tetris display (for 5 second duration). During the initial 12 min of the competition, five “neutral” messages (e.g. “Do your best”, “Go, go, go”) were presented (every 2 min). These messages were identical across conditions and did not imply relative performance of the two competitors. During the final 3 min of the contest, two further messages were displayed that were condition specific:

Clear winners: “Keep going, you are far ahead!”/“Come on, you are far in the lead!”

Clear losers: “Keep going, you are far behind!”/“Come on, you are far behind!”

Narrow winners: “Keep going, you are only just in front!”/“Come on, you are only just ahead!”

Narrow losers: “Keep going, you are slightly behind!”/“Come on, you are only just behind!”.

The purpose of these divergent messages was to prepare the participant for the impending outcome, in order to maximize the degree of testosterone change and our ability to detect that change with a single saliva sample at 20 min post-competition (Zilioli and Watson, 2012). These feedback messages also model aspects of real-world competitions in which competitors have regular feedback about their position relative to their opponents. Note that this approach in which performance feedback was provided during the competition aligns with Study 1 of Zilioli et al. (2014) but is different from Study 2 of Zilioli et al. (2014) in which no performance feedback was given.

### 2.3 Procedure

#### 2.3.1 Pre-competition phase

Upon arriving, each pair of competitors were greeted by a male experimenter, and each participant was led to one of two adjacent test rooms, where they completed a consent form, a demographic questionnaire, and the Positive and Negative Affect Schedule (PANAS; Watson et al., 1988) to assess baseline mood state. Participants were given instructions to the Tetris game. To intensify the competition, participants were then instructed that the Tetris winner would receive a trophy engraved with the text “Tetris Winner” and a chocolate bar. The opponents then met each other again in the corridor outside the test rooms. They commenced the game on the experimenter's instruction, at which point the doors to the two testing rooms were shut for the duration of the testing session. The two testing rooms were soundproof, so that participants were not aware of the progress of the opponent in the other room.

#### 2.3.2 Post-competition phase

After competing for 15 min, the on-screen feedback was displayed. At this point, the doors to the two testing rooms were opened and the experimenter entered the testing room of the winner, announcing within earshot of the opponent “Congratulations, you won!”. The experimenter then entered the testing room of the loser, and announced loudly “Sorry, you lost”. Following the competition, participants were asked to give a closeness rating (“How close was the result of the game relative to your partner?”: 1 = *extremely far away* to 9 = *extremely close*), a pleasantness rating (“How pleased were you with the Tetris outcome?”: 1 = *extremely unpleased* to 9 = *extremely pleased*), and a motivation rating (“How much do you want to continue playing the Tetris game”: 1 = *not at all* to 9 = *very much*). They also completed the PANAS for a second time.

### 2.4 Saliva samples and hormone assays

To reduce diurnal hormone variability, all testing occurred between 13:00 h and 19:00 h (Campbell et al., 1982). After completing informed consent, a demographic questionnaire and PANAS, participants provided a baseline saliva sample (t_0_). They started Tetris competition 5 min (t_5_) after the collection of the t_0_ saliva sample. After completing the competition and revealing the winner and loser (t_20_), participants completed the post-experiment questionnaire and then viewed a neutral video clip (a documentary about Ireland, serving as filler task) in their own test rooms while doors being closed again. At exactly 20 min after the finishing of the Tetris competition (t_40_), participants provided a second saliva sample and then completed some cognitive tasks assessing dominance behavior (not reported here). Participants were then debriefed as to the rigged nature of the outcome and were paid the participant fees.

Participants were instructed to abstain from eating, drinking, smoking, or brushing their teeth for 1 h before testing. Saliva samples were collected using passive drool (Salimetrics, Suffolk, England). Samples were chilled immediately following collection, and then frozen within 1 h and held at − 80° until assay. Samples were assayed in duplicate using competitive enzyme immunoassays for testosterone and cortisol (Salimetrics, Suffolk, England). The average intra-assay coefficient of variation was 2.04% for testosterone and 2.16% for cortisol, and inter-assay coefficients averaged across high and low controls were 4.98% for testosterone and 4.46% for cortisol.

### 2.5 Statistical analysis

The average Tetris score was 3935.30 points (*SD* = 2891.27), in a similar range with a previous report using the same task (Zilioli et al., 2014). Two participants had extreme low scores on the Tetris task (scoring less than 200 points) and were excluded. One participant reported having major depression and was taking medication, and one participant was found to have participated in the behavioral validation study (see Supplementary material). Analysis excluded these four participants. Tetris scores and the subjective ratings were analyzed using ANOVA with Outcome (win vs. loss) and Closeness (narrow vs. clear) as the two between-subjects factors.

For the hormone data, one participant had blood contamination in the saliva samples, and one participant did not provide sufficient saliva sample, leaving a sample of 160 participants for hormone assessment. Baseline (T0) and post-competition testosterone (T1) concentrations were normally distributed. Cortisol values for one participant and testosterone values for 3 participants differed by more than three standard deviations from the normalized means and were excluded. Testosterone unstandardized residuals, obtained by regressing post-competition testosterone (T1) against baseline testosterone (T0), were used as a measure of testosterone change, as described in Zilioli and Watson (2012). Cortisol but not testosterone levels were log transformed. Bonferroni correction was applied to multiple comparisons.

## 3 Results

### 3.1 Behavioral analysis

For the Tetris overall scores, neither the main effect of Outcome nor Closeness were significant, both *p*s > 0.1. There was no significant interaction between Outcome and Closeness, *p* > 0.1. Thus the conditions did not differ in the objective performance on the Tetris game.

On the ratings of closeness, a 2 (Outcome: win vs. loss) × 2 (Closeness: narrow vs. clear) ANOVA revealed a significant main effect of Closeness, *F*(1,158) = 2420.80, *p* < 0.001, η_p_^2^ = 0.94, with participants in the narrow condition (*M* = 8.63, *SD* = 0.66) giving higher closeness ratings than participants in the clear condition (*M* = 2.53, *SD* = 0.90). Neither the main effect of Outcome nor the interaction term were significant, both *p*s > 0.1.

On the PANAS, winning (*M* = 3.92, *SD* = 6.04) increased positive affect relative to losing (*M* = − 2.30, *SD* = 5.45), *F*(1,158) = 46.60, *p* < 0.001, η_p_^2^ = 0.23. Wins (*M* = − 1.57, *SD* = 4.52) decreased negative affect compared to losses (*M* = 0.57, *SD* = 4.73), *F*(1,158) = 8.31, *p* < 0.01, η_p_^2^ = 0.05. Closeness did not influence positive affect or negative affect, nor were the interactions with Outcome significant, all *p*s > 0.1. Likewise, for the more specific pleasantness rating (referring directly to the Tetris result), wins were rated as more pleasant than losses, *F*(1,158) = 279.02, *p* < 0.001, η_p_^2^ = 0.64. There was also a significant main effect of Closeness, *F*(1,158) = 6.35, *p* = 0.01, η_p_^2^ = 0.039, and a significant Outcome × Closeness interaction, *F*(1,158) = 9.97, *p* < 0.01, η_p_^2^ = 0.059. Simple effect tests showed that clear winners (*M* = 7.35, *SD* = 1.37) and narrow winners (*M* = 7.21, *SD* = 1.32) did not differ in pleasantness rating, *p* > 0.1, but for the losers, narrow losses (*M* = 4.23, *SD* = 1.66) were experienced as less unpleasant than clear losses (*M* = 2.97, *SD* = 1.22), *t* (77) = − 3.81, *p* < 0.001, *d* = 0.86.

On the motivation rating, neither main effect of Outcome nor Closeness were significant, both *p*s > 0.1. There was no significant interaction between Outcome and Closeness, *p* > 0.1.

### 3.2 Hormone responses

#### 3.2.1 Preliminary analysis

Descriptive statistics for baseline and post-competition testosterone levels, and untransformed cortisol levels are presented in Table 1.

**Table 1:** Descriptive statistics for raw hormone measures. SEM = standard error of the mean.

	All participants (n = 156)		Clear winners (n = 39)		Narrow winners (n = 40)		Clear losers (n = 38)		Narrow loser (n = 39)	
	*M* (*SEM*)	SD	*M* (*SEM*)	SD	*M* (*SEM*)	SD	*M* (*SEM*)	SD	*M* (*SEM*)	SD
Pre-competition testosterone (pg/mL)	150.31 (3.27)	40.87	148.28 (6.87)	42.93	143.93 (6.09)	38.49	154.27 (6.74)	41.52	155.01 (6.58)	41.07
Post-competition testosterone (pg/mL)	150.80 (3.22)	40.18	150.89 (6.82)	42.59	139.41 (5.39)	34.11	156.04 (6.85)	42.26	157.28 (6.47)	40.41
Change in testosterone (pg/mL)^a^	0.49 (2.02)	25.17	2.61 (4.05)	25.32	− 4.52 (3.66)	23.14	1.77 (4.89)	30.15	2.27 (3.48)	21.75
Pre-competition cortisol (μg/dL)^b^	0.18 (0.008)	0.10	0.17 (0.01)	0.08	0.18 (0.02)	0.11	0.21 (0.022)	0.13	0.17 (0.01)	0.09
Post-competition cortisol (μg/dL)^c^	0.17 (0.008)	0.10	0.17 (0.02)	0.10	0.14 (0.01)	0.07	0.20 (0.02)	0.10	0.15 (0.02)	0.10

a Post-competition testosterone minus baseline testosterone.

b Means, standard deviation and standard error of the mean (SEM) were calculated from the untransformed baseline cortisol distribution.

c Means, standard deviation and standard error of the mean (SEM) were calculated from the untransformed post-competition cortisol distribution.

Consistent with previous findings (Popma et al., 2007, Zilioli and Watson, 2012), testosterone and log-transformed cortisol levels were moderately positively correlated, *r* = 0.43, *p* < 0.001. Prior to Tetris competition, baseline testosterone and cortisol levels did not differ between experimental conditions, all *p*s > 0.1, and thus data are appropriate for conversion to change from baseline values. Time of the day did not correlate with baseline testosterone, *p* > 0.1, and hence was not included in the analysis.

#### 3.2.2 Competition effect

**Fig. 1:**
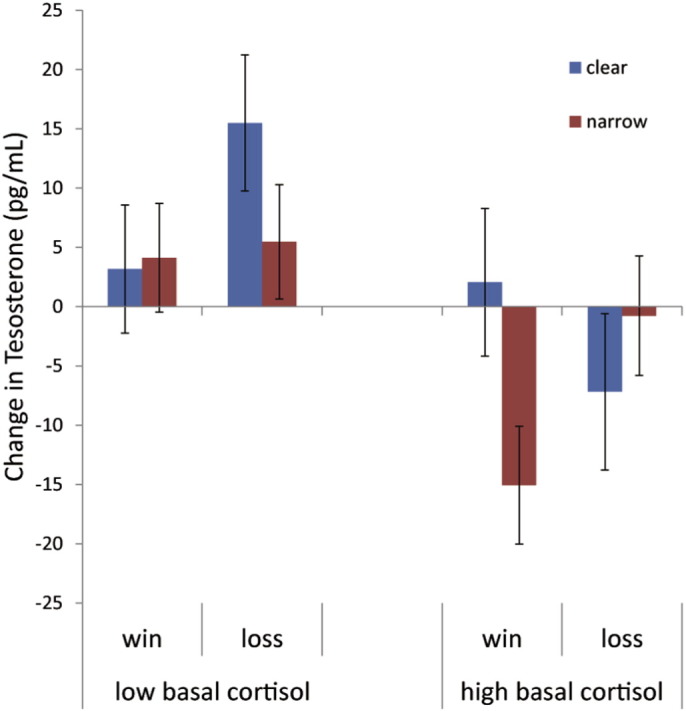
Acute changes in testosterone (post-competition testosterone minus pre-competition testosterone) to the competition outcomes in participants separated on basal cortisol levels using a median split. Error bars represent standard errors of the mean. Number of participants in each condition: for low basal cortisol group, clear wins (n = 20), narrow wins (n = 22), clear losses (n = 15), and narrow losses (n = 19); for high basal cortisol group, clear wins (n = 19), narrow wins (n = 18), clear losses (n = 23), and narrow losses (n = 20).

A preliminary model examined the effect of the competition outcomes on hormone responses was examined using a 2 (Outcome: win vs. loss) × 2 (Closeness: narrow vs. clear) ANOVA. For the testosterone unstandardized residuals, neither main effect terms nor the Outcome × Closeness interaction were significant, all *p*s > 0.1. Given testosterone responses depend on basal cortisol levels (Edwards and Casto, 2015, Zilioli and Watson, 2012), we explored the extent to which basal cortisol interacted with competition outcome and closeness to predict testosterone changes. Participants were separated into high and low cortisol groups using a median split on the baseline cortisol distribution.^2^ There was a significant 3-way interaction between Outcome, Closeness and Basal Cortisol group on the testosterone change, *F*(1,148) = 5.65, *p* = 0.019, η_p_^2^ = 0.037. The interaction was decomposed by looking at the effects of Outcome and Closeness among the high and low basal cortisol subgroups separately (see Fig. 1). For the low basal cortisol group, neither main effect terms nor the interaction term were significant, all *p*s > 0.1. For the high basal cortisol group, there was significant interaction between Outcome and Closeness, *F*(1,76) = 5.20, *p* = 0.025, η_p_^2^ = 0.06. Narrow wins (*M* = − 17.00, *SD* = 20.44) reduced testosterone levels (− 9.03%, in terms of change ratio) compared to clear wins (*M* = 1.36, *SD* = 24.40), *t* (35) = 2.47, *p* = 0.018, *d* = 0.82), but there was no difference between clear losses (*M* = − 8.00, *SD* = 30.49) and narrow losses (*M* = − 1.03, *SD* = 20.81), *p* > 0.1. Narrow wins also reduced testosterone levels compared to narrow losses, *t*(36) = − 2.38, *p* = 0.023, *d* = 0.77. These findings were corroborated by testing correlations between basal cortisol and testosterone change within each experimental condition, such that there was significant negative correlation between basal cortisol and testosterone change for narrow wins (see Table 2).

**Table 2:** Correlations between basal cortisol (log-transformed) and testosterone change within each experimental condition.

	Clear *r*	Narrow *r*
Winners	− 0.02	− 0.37⁎
Losers	− 0.28	− 0.23

⁎ *p* < 0.05.

We analyzed the pleasantness and motivation subjective ratings as dependent variables in a model using Outcome (win vs. loss) × Closeness (clear vs. narrow) × Basal Cortisol (high vs. low); both 3-way interactions were non-significant, *p*s > 0.1. Testosterone changes were not correlated with motivation ratings for each condition, *p*s > 0.1.

## 4 Discussion

Competitors who experienced narrow outcomes perceived these outcomes as closer to their opponent than those who experienced clear outcomes, showing that our 2-player Tetris competition game can be used to investigate the psychological processing of near events. Closeness also modulated outcome appraisals, such that narrow losses were experienced as more pleasant than clear losses, although neither outcome, closeness, nor their interaction influenced mood or motivation. This is consistent with previous reports that the performance feedback of being slightly behind an opponent (Berger and Pope, 2011) or just failing to obtain a reward (Wadhwa and Kim, 2015) can induce positive emotional effects. These findings generalize some of the psychological responses (i.e. increased motivation to play) seen after gambling near-misses (Clark et al., 2009) to a broader context of social, competitive interactions.

Winning or losing the competition had no reliable overall effect on testosterone fluctuations, in contrast to the classical “winner–loser effect” (Mazur, 1985, Mazur and Booth, 1998). However, testosterone responses to our social competition depended on whether the outcomes were clear or narrow, and were further modulated by basal cortisol levels. These observations are consistent with the broad recognition that various psychological and physiological factors moderate the winner-loser effect (Carré and Olmstead, 2015). Specifically, we saw that narrowly winning a competition decreased testosterone levels among individuals with higher basal cortisol levels. Although the effect size for this finding was small, it extends the experiment by Zilioli et al. (2014) comparing testosterone responses to narrow wins and losses without the inclusion of the decisive (clear win/clear loss) conditions. That study demonstrated a similar reversal in the testosterone response in a female sample, such that narrow losers displayed testosterone increases relative to narrow winners, in competitions where the performance feedback was either uncertain or ambiguous. Like Zilioli et al. (2014), our effect was also largely driven by a decrease in testosterone in the narrow win condition. The status instability hypothesis proposes that within unstable social hierarchies, decreased testosterone concentrations may drive avoidance of further competitive encounters in individuals with insecure social status (i.e. determined by the outcome of the competition), in order to protect their now-vulnerable social rank. The present study tested the status instability hypothesis more directly, using a 2 × 2 fully factorial design, in a relatively large sample of male participants. These findings indicate that in individuals with high baseline cortisol levels, competitions in which the outcome is a neck-to-neck finish may render the status hierarchy unstable, such that the narrow winners appraise an objective victory as a more ambiguous outcome.

Emotional responses to competitive outcomes are critically influenced by cognitive appraisals including perceived control and attributions of skill versus chance (Biddle and Hill, 1988). Likewise, testosterone reactivity does not respond simply to winning or losing outcomes in a categorical manner (see Salvador and Costa, 2009 for a review). In a study of basketball players, testosterone changes did not differ between winners and losers, but correlated with the ratio of the score to the time spent playing, reflecting each individual's contribution to the overall team outcome (Gonzalez-Bono et al., 1999). External attributional styles moderate the testosterone change: the tendency to attribute the team's success to external factors (i.e. luck and chance) buffered individual testosterone increases (Gonzalez-Bono et al., 1999, Gonzalez‐Bono et al., 2000), whereas players who attributed their victories to skill showed the strongest testosterone increase (Gonzalez-Bono et al., 2000). In our Tetris competition, the narrow winners finished just 11 points ahead, and this tight difference may trigger an attribution of success to chance factors rather than personal skill – particularly in an unpracticed task where the participants were naïve to their opponents' ability level. Such appraisals might underlie the observed decreases in testosterone in the narrow win condition. Future studies may fruitfully measure such attributions directly, and test the effects of manipulating practice or perceived opponent skill to further explore these possibilities.

The observed testosterone decrease among individuals high in basal cortisol also merges with the well-established effect of elevated cortisol levels in depression and other mood disorders (Burke et al., 2005, Kirschbaum and Hellhammer, 1994). Indeed, a supplementary analysis in our sample confirmed a relationship between basal cortisol and negative affect, *r* = 0.21, *p* < 0.01. Depression is also associated with changes in the processing of ambiguous feedback. While healthy individuals display typical self-serving biases in appraising control over positive outcomes (and denial of personal involvement in negative outcomes), depressed individuals can be more accurate in appraising their lack of control over chance outcomes (Alloy and Abramson, 1979). Our observation that the effects of narrow wins on testosterone levels were restricted to participants with relatively high basal cortisol can be interpreted in relation to these effects: the hypothalamic-pituitary-adrenal (HPA) axis may mediate the tendency to attribute ambiguous events to personal skill versus chance. Future studies could test the effects of mood or stress on the processing of near outcomes.

Our results also speak to recent findings that the hypothalamic-pituitary-gonadal (HPG) axis and HPA axis jointly regulate dominance behavior (the “dual hormone hypothesis”, Mehta and Josephs, 2010, Mehta et al., 2015; see Mehta and Prasad, 2015 for a review). There is increasing recognition that there is mutual influence of the HPG and HPA axis on each other (Viau, 2002). In past studies, testosterone is positively correlated with status-seeking behaviors only when basal cortisol levels are low, and testosterone's effect on status-seeking behavior is inhibited in individuals with high basal cortisol (Mehta and Josephs, 2010). Zilioli and Watson (2012) extended this hypothesis by showing that testosterone responses to competition outcomes depend on both basal testosterone levels and basal cortisol levels, such that the winner-loser effect was strongest in individuals with high testosterone and low cortisol levels. Our findings add to this body of work highlighting the importance of measuring HPA function when studying testosterone responses in social contests. Future work should build upon this work by testing not only the moderating role of basal cortisol but also the interaction between basal testosterone and cortisol on testosterone changes (Mehta and Josephs, 2010, Zilioli and Watson, 2012).

Some limitations of the study should be noted. Our experiment tested a large group of male participants, and while we replicated a reversed winner-loser effect described by Zilioli et al. (2014) in a female sample, future studies would benefit from including both genders in the same design to enable direct comparisons to be tested. Second, our test for the modulatory effect of basal cortisol used a median split. There are drawbacks to this approach including loss of statistical power (Aiken and West, 1991), although we obtained essentially the same pattern of results by treating basal cortisol as a continuous variable (see footnote 2). Third, the predictions of the status instability hypothesis were only partially supported in our data, given that narrow losses should *also* destabilize the status hierarchy, driving testosterone fluctuation. Our findings were limited to the narrow win condition, and such changes to narrow loss were also absent in the study by Zilioli et al. (2014). The status instability hypothesis was supported only in high basal cortisol individuals but not low basal cortisol individuals, which we did not predict in advance. Future studies are needed to characterize boundary conditions of the status instability hypothesis. Fourth, in our study, the messages that were displayed to the participants made it such that participants' expectations matched with the eventual outcome, whereas no performance feedback was given in the Study 2 of Zilioli et al. (2014). Zilioli et al. (2014) argued that one of the potential mechanisms through which the reversed winner-loser effect occurs is through expectancy violations that lead to feelings of surprise especially in uncertain or close losers. We would encourage further work testing the modulatory role of prediction errors in the status instability hypothesis. Fifth, testosterone changes were not correlated with self-reported motivation to play in the present study, future research that measures observable status-seeking behaviors in actual social interactions or that includes more saliva samples may detect connections between testosterone fluctuations and future behavior.

To conclude, we found that narrow losses in a competition elicited a positive emotional effect. Narrow wins decreased testosterone concentrations among individuals higher in basal cortisol, consistent with the emerging literature on the interaction between HPG and HPA axes. This finding is interpreted within the status instability hypothesis and highlights the significance of clear versus narrow outcomes in moderating testosterone fluctuations.

## Funding statement

This project was supported by Medical Research Council (MRC Ref G1000183) and Wellcome Trust (WT Ref 093875/Z/10/Z). The funding sources had no further role in study design, data collection, analysis, interpretation, or decision to submit this manuscript for publication.

## Conflict of interest statement

Dr. Clark: The Centre for Gambling Research at UBC is supported by funding from the British Columbia Lottery Corporation and the Province of British Columbia.

The other authors declare that they had no conflicts of interest with respect to their authorship or the publication of this article.

## Appendix A Supplementary data

Supplementary material. Image 1

## Footnotes

1 In a competition with a binary outcome (i.e. win vs. loss), a “narrow win” could alternatively be termed a “near loss”. Conversely a “narrow loss” could alternatively be termed a “near win”. For ease of clarity in labelling the cells of our 2 × 2 design, “narrow” vs. “clear” were used here.

2 We also explored the influence of basal cortisol by entering it as a continuous variable. Data were winsorized by 2 SD to correct for skew and outliers. In a linear regression model with Outcome and Closeness as categorical predictors and basal cortisol as a continuous predictor, the three-way interaction on testosterone change was marginally significant, *b* = − 18.11, *SE* = 15.08, *t* = − 1.20, *p* = 0.08, η_p_^2^ = 0.04.

## References

[bb0005] Aiken L.S., West S.G. (1991). Multiple Regression: Testing and Interpreting Interactions.

[bb0010] Alloy L.B., Abramson L.Y. (1979). Judgment of contingency in depressed and nondepressed students: sadder but wiser?. J. Exp. Psychol. Gen..

[bb0015] Archer J. (2006). Testosterone and human aggression: an evaluation of the challenge hypothesis. Neurosci. Biobehav. Rev..

[bb0020] Berger J., Pope D. (2011). Can losing lead to winning?. Manag. Sci..

[bb0025] Biddle S.J.H., Hill A.B. (1988). Causal attributions and emotional reactions to outcome in a sporting contest. Personal. Individ. Differ..

[bb0030] Burke H.M., Davis M.C., Otte C., Mohr D.C. (2005). Depression and cortisol responses to psychological stress: a meta-analysis. Psychoneuroendocrinology.

[bb0035] Campbell I.C., Walker R.F., Riad-Fahmy D., Wilson D.W., Griffiths K. (1982). Circadian rhythms of testosterone and cortisol in saliva: effects of activity-phase shifts and continuous daylight. Chronobiologia.

[bb0040] Carré J.M., Olmstead N. (2015). Social neuroendocrinology of human aggression: examining the role of competition-induced testosterone dynamics. Neuroscience.

[bb0045] Carré J.M., Campbell J.A., Lozoya E., Goetz S.M.M., Welker K.M. (2013). Changes in testosterone mediate the effect of winning on subsequent aggressive behaviour. Psychoneuroendocrinology.

[bb0050] Clark L., Lawrence A.J., Astley-Jones F., Gray N. (2009). Gambling near-misses enhance motivation to gamble and recruit win-related brain circuitry. Neuron.

[bb0055] Cote D., Caron A., Aubert J., Desrochers V., Ladouceur R. (2003). Near wins prolong gambling on a video lottery terminal. J. Gambl. Stud..

[bb0060] Edwards D.a., Casto K.V. (2015). Baseline cortisol moderates testosterone reactivity to women's intercollegiate athletic competition. Physiol. Behav..

[bb0065] Gonzalez-Bono E., Salvador A., Serrano M.A., Ricarte J. (1999). Testosterone, cortisol, and mood in a sports team competition. Horm. Behav..

[bb0070] Gonzalez‐Bono E., Salvador A., Ricarte J., Serrano M.A., Arnedo M. (2000). Testosterone and attribution of successful competition. Aggress. Behav..

[bb0075] Hamilton L.D., Carré J.M., Mehta P.H., Olmstead N., Whitaker J.D. (2015). Social neuroendocrinology of status: a review and future directions. Adapt. Hum. Behav. Physiol..

[bb0080] Kirschbaum C., Hellhammer D.H. (1994). Salivary cortisol in psychoneuroendocrine research: recent developments and applications. Psychoneuroendocrinology.

[bb0085] Magee J.C., Galinsky A.D. (2008). 8 social hierarchy: the self‐reinforcing nature of power and status. Acad. Manag. Ann..

[bb0090] Mazur A. (1985). A biosocial model of status in face-to-face primate groups. Soc. Forces.

[bb0095] Mazur A., Booth A. (1998). Testosterone and dominance in men. Behav. Brain Sci..

[bb0100] Mehta P.H., Josephs R.A. (2006). Testosterone change after losing predicts the decision to compete again. Horm. Behav..

[bb0105] Mehta P.H., Josephs R.A. (2010). Testosterone and cortisol jointly regulate dominance: evidence for a dual-hormone hypothesis. Horm. Behav..

[bb0110] Mehta P.H., Prasad S. (2015). The dual-hormone hypothesis: a brief review and future research agenda. Curr. Opin. Behav. Sci..

[bb0115] Mehta P.H., Mor S., Yap A.J., Prasad S. (2015). Dual-hormone changes are related to bargaining performance. Psychol. Sci..

[bb0120] Oliveira G.A., Uceda S., Oliveira T., Fernandes A., Garcia-Marques T., Oliveira R.F. (2013). Threat perception and familiarity moderate the androgen response to competition in women. Front. Psychol..

[bb0125] Oliveira G.A., Uceda S., Oliveira T.F., Fernandes A.C., Garcia-Marques T., Oliveira R.F. (2014). Testosterone response to competition in males is unrelated to opponent familiarity or threat appraisal. Front. Psychol..

[bb0130] Popma A., Vermeiren R., Geluk C.a.M.L., Rinne T., van den Brink W., Knol D.L., Jansen L.M.C., van Engeland H., Doreleijers T.a.H. (2007). Cortisol moderates the relationship between testosterone and aggression in delinquent male adolescents. Biol. Psychiatry.

[bb0135] Salvador A., Costa R. (2009). Coping with competition: neuroendocrine responses and cognitive variables. Neurosci. Biobehav. Rev..

[bb0140] Sapolsky R.M. (2004). Social status and health in humans and other animals. Annu. Rev. Anthropol..

[bb0145] Viau V. (2002). Functional cross-talk between the hypothalamic-pituitary-gonadal and -adrenal axes. J. Neuroendocrinol..

[bb0150] Wadhwa M., Kim J.C. (2015). Can a near win kindle motivation? The impact of nearly winning on motivation for unrelated rewards. Psychol. Sci..

[bb0155] Watson D., Clark L.A., Tellegen A. (1988). Development and validation of brief measures of positive and negative affect: the PANAS scales. J. Pers. Soc. Psychol..

[bb0160] Zilioli S., Watson N.V. (2012). The hidden dimensions of the competition effect: basal cortisol and basal testosterone jointly predict changes in salivary testosterone after social victory in men. Psychoneuroendocrinology.

[bb0165] Zilioli S., Watson N.V. (2014). Testosterone across successive competitions: evidence for a “winner effect” in humans?. Psychoneuroendocrinology.

[bb0170] Zilioli S., Mehta P., Watson N.V. (2014). Losing the battle but winning the war: uncertain outcomes reverse the usual effect of winning on testosterone. Biol. Psychol..

