# Supplementary material for: Comparison of clear and narrow outcomes on testosterone levels in social competition
Source: Horm Behav. 2017 Jun;92:51–6. doi: 10.1016/j.yhbeh.2016.05.016 (PMC5726082; doi:10.1016/j.yhbeh.2016.05.016)
Supplement: Supplementary file 1 — Supplementary material. [file mmc1.docx]

**Supplementary Material: Behavioral Validation Study**

The aim of the pilot study was to establish the sensitivity of a modified version of the Tetris competition task to clear vs. narrow outcomes. The task and procedure were identical to the main report, with the exception that in the absence of testosterone sampling, we recruited both males and females.

**Methods**

*Participants*

Eighty-seven volunteers (32 male, 55 female; mean age = 27.4, SD = 4.50; age range = 19 – 40) were recruited through university advertisements. Participants attended a single testing session in same-sex pairs, where they completed the Tetris game (15 min) and post-experiment questionnaires. In cases where only one participant signed up for a testing slot (N = 20), this participant was paired with a same-sex confederate; exploratory analyses were run comparing the participants who were paired with a confederate versus an actual participant; this factor did not impact any of the dependent variables, and thus was omitted in the Results section. Participants were reimbursed £8 for their participation.

*Statistical Analysis*

The average Tetris score was 3332.09 points (SD = 2529.56), which is in a similar range to a previous report using the same task (Zilioli et al., 2014). Six participants performed extremely poor in the task (scoring less than 200 points) such that a rigged clear win would not be convincing. These participants were excluded; their inclusion does not change the ordinal pattern of results. Gender did not have any significant main effect nor interact with any other variables, thus we combined them together. Subjective ratings were analysed using ANCOVA with Outcome (win vs. loss) and Closeness (narrow vs. clear) as two independent variables, and Tetris score as a covariate (given an inadvertent difference in Tetris scores between conditions, see below).

**Results and discussion**

On the overall Tetris scores, there was a significant main effect of Outcome, *F*(1,77) = 4.43, *p* = .039, η_p_^2^ = .05, such that the (assigned) winners (*M* = 4135.08, *SD* = 2422.01) did actually achieve more points than the (assigned) losers (*M* = 3003.46, *SD* = 2418.55). Neither the main effect of Closeness nor the interaction term were significant, *p*s > .1. Therefore, Tetris score was added as a covariate in the analysis of the subjective ratings.

On the rating of “How close was the result of the game relative to your partner”, there was a significant main effect of Closeness, *F* (1, 76) = 420.84, *p* < .001, η_p_^2^ = 0.85. Participants with narrow outcomes (*M* = 8.47, *SD* = 0.76) perceived their scores as closer to their opponent, compared to those groups experiencing clear outcomes (*M* = 2.77, *SD* = 1.52). Neither the main effect of Outcome nor the interaction term were significant, *p*s > .1. The covariate (actual Tetris score) was not significantly related to perceived closeness, *p* > .1.

On the PANAS^[[1]](#footnote-1)^, winning increased positive affect (*M* = 3.29, *SD* = 7.21) compared to losing (*M* = –3.32, *SD* = 7.22), *F*(1,75) = 16.30, *p* < .001, η_p_^2^ = 0.18, and decreased negative affect (*M* = –2.50, *SD* = 4.95) compared to losing (*M* = –0.54, *SD* = 4.95), *F*(1,75) = 3.04, *p* = 0.085, η_p_^2^ = 0.04. Closeness did not influence either positive affect or negative affect nor interact with Outcome, all *p*s > .1. The covariate, the actual Tetris score, was not significantly related to either positive or negative affect, both *p*s > .1.

On the outcome-specific rating of “How pleased were you with the Tetris outcome?”, there was a significant main effect of Outcome (*F*(1,76) = 94.70, *p* < .001, η_p_^2^ = 0.56), confirming enhanced pleasantness ratings following wins. Using this more directive question, there was a significant Outcome × Closeness interaction, *F*(1,76) = 5.86, *p* = .018, η_p_^2^ = 0.07. The covariate, actual Tetris score, was not significantly related to pleasantness ratings, *p* > .1. The interaction term was decomposed by looking at the effect of Closeness on winning and losing outcomes separately. For wins, there was no reliable difference between clear winners (*M* = 7.36, *SD* = 1.47) and narrow winners (*M* = 6.84, *SD* = 1.34), *p* > .1. For losers, narrow losses (*M* = 4.37, *SD* = 1.86) were significantly less aversive than clear losses (*M* = 3.33, *SD* = 1.28), *F*(1,37) = 5.48, *p* = .25, η_p_^2^ = 0.13.

On the motivation rating (i.e. “How much do you want to continue playing the Tetris game?”), the 2 (Outcome: win vs. loss) × 2 (Closeness: narrow vs. clear) ANCOVA also revealed a significant Outcome × Closeness interaction, *F* (1,76) = 4.29, *p* < .05, η_p_^2^ = 0.05. Neither the main effect of Outcome nor Closeness was significant, *p*s > .1. The Tetris score covariate was not significantly related to the motivation rating, *p* > .1. To decompose the interaction effect, we looked at the effect of Closeness on wins and losses separately. For winners, there was no difference between clear winners (*M* = 6.64, *SD* = 1.71) and narrow winners (*M* = 6.37, *SD* = 1.67), *p* > .1. For losers, narrow losses (*M* = 7.37, *SD* = 1.74) were associated with a greater motivation to continue than clear losses (*M* = 5.90, *SD* = 2.07), *F* (1, 37) = 5.89, *p* = .02, η_p_^2^ = 0.14. The narrow losers also showed a trend of increased motivation compared to narrow winners, *F* (1, 35) = 3.59, *p* = .066, η_p_^2^ = 0.09, whereas there was no difference between clear winners and clear losers, *p* > .1.

In summary, our manipulation check suggests that the Tetris task successfully induced differential feelings of closeness, such that competitors who experienced narrow outcomes perceived the outcomes as closer relative to those who experienced the clear outcomes. Outcome closeness did not modulate general positive or negative mood, but it did influence the specific appraisal of the competition outcome: narrow losses were experienced as more pleasant than clear losses. Narrow losses also increased self-reported motivation, compared to both narrow wins and clear losses, corroborating previous findings that being slightly behind in a competition has a positive motivational effect (Berger and Pope, 2011).

1. One participant’s PANAS data was missing due to technical failure. [↑](#footnote-ref-1)
